# Clinical benefit of high dose IL-2 (HD IL-2) therapy: evidence for improved overall survival in patients with stable disease

**DOI:** 10.1186/2051-1426-1-S1-P105

**Published:** 2013-11-07

**Authors:** Tasha Hughes, Gail Iodice, Sanjib Basu, Steven Bines, Howard Kaufman

**Affiliations:** 1 Rush University Medical Center, Chicago, IL, USA; 2 Columbia University Medical Center, New York, NY, USA

## Background

HD IL-2 is an approved immunotherapy for advanced melanoma and renal cell carcinoma with a reported objective response rate (ORR) up to 20%. Patients with stable disease (SD) following treatment are not considered objective responders and thus are not included in reported response rates. Since the initial reports of IL-2 our understanding of the delayed pharmacokinetics of immunotherapy has improved. The purpose of this study is to report the clinical outcomes for patients with stable response to HD IL-2.

## Methods

A retrospective chart review of 305 patients diagnosed with metastatic renal cell carcinoma or melanoma and treated with HD IL-2 therapy over a 12-year period was conducted. Age, response to HD IL-2 (based on RECIST criteria) and survival data were available in 215 patients at the time of analysis. Survival analysis was conducted on all patients for whom complete data was available and Kaplan-Meier curves were generated based on overall survival.

## Results

Among the 215 patients with complete data, 62% had progressive disease (PD), 24% had SD and the objective response rate was 14%. Median overall survival was 16.8 months among all 215 patients. Patients with a PR or CR (median survival not reached in the study period) had significantly prolonged survival than the remaining patients (median survival 13.9 months, log-rank p < 0.01). Patients with SD (median survival of 38.2 months) had improved survival compared to patients with progressive disease (median survival of 7.9 months, log-rank p< 0.01).

## Discussion

The IL-2 literature has focused on partial or complete responders when reporting objective response rates. In our data, as well as elsewhere in the literature, there are a large number of patients who have a stable response to HD IL-2 that have not been included in the ORR. Our data supports a significant survival benefit associated with stable disease following IL-2, which suggests that HD IL-2 may result in a clinical benefit in a larger number of patients than has previously been reported. Consideration of a disease control rate (DCR) inclusive of objective responders and stable responses may be more appropriate to better define the clinical benefit of IL-2.

